# Multiple Hepatic Abscesses Secondary to Streptococcus anginosus Infection: A Case Report and Review of the Literature

**DOI:** 10.7759/cureus.28415

**Published:** 2022-08-25

**Authors:** Bhesh R Karki, Louis Costanzo, Suman K Jha, Steffi Nainan, Samy I. McFarlane

**Affiliations:** 1 Internal Medicine, Downstate-Health Science University, Brooklyn, USA; 2 Internal Medicine, University of Louisville, Louisville, USA; 3 Medicine, Division of Endocrinology, Downstate-Health Science University, Brooklyn, USA

**Keywords:** gastrointestinal flora, streptococcus milleri, streptococcus anginosus, pyogenic liver abscess, hepatic abscess

## Abstract

Hepatic abscesses are rare and generally present as solitary lesions in immunocompromised patients. The development of multiple hepatic abscesses in an immunocompetent patient is relatively uncommon. We report a rare case of a 73-year-old woman who presented with fever and right upper quadrant abdominal tenderness. Laboratory findings were significant for leukocytosis, transaminitis, and elevated inflammatory markers. Peripheral blood culture grew Streptococcus anginosus. Computed tomography of the abdomen and pelvis (CT A/P) revealed multiple hypoattenuating ill-defined cystic lesions in the liver consistent with abscesses formation; this was confirmed by magnetic resonance cholangiopancreatography (MRCP). The patient underwent appropriate treatment with antibiotics. Upon a three-week follow-up, the patient’s symptoms subsided, and her laboratory parameters normalized. Although Streptococcus anginosus is a normal gastrointestinal flora, it has the potential to form abscesses. Our report indicates the importance of considering Streptococcus anginosus in the differential diagnosis. Management includes four to six weeks of antibiotic therapy together with drainage of larger abscesses.

## Introduction

A pyogenic liver abscess (PLA) is a highly fatal condition, and the main cause of mortality is septic shock [1]. Early use of imaging techniques like computed tomography (CT) scan and magnetic resonance imaging (MRI) helps in the timely diagnosis of the disease and better prognosis. The challenge remains yet to find the source of the infection causing a liver abscess. Biliary tract disorder, systemic infections (infective endocarditis, pyelonephritis), and colonic disorders are the major sources and need to be adequately investigated [2].

We report a rare case of an immunocompetent female with multiple liver abscesses caused by Streptococcus anginosus, which was managed successfully with antibiotics.

## Case presentation

A 73-year-old woman with well-controlled type II diabetes mellitus, hyperlipidemia, and osteoporosis presented to the emergency department with complaints of fever associated with chills, watery diarrhea (4-5 episodes/day), and diffuse abdominal discomfort for 2-3 days. She denied having myalgia, nausea/vomiting, yellowish discoloration of eyes, burning micturition, chest pain, air hunger, recent travel or eating outside, and recent instrumentation. The patient had a history of appendectomy as a child and an ectopic pregnancy surgery in the distant past.

On physical examination, the patient had a temperature of 102.7 F (oral), heart rate of 137/min, respiratory rate of 20/min, and her blood pressure was measured to be 96/58 mm of Hg. The abdomen was soft with mild tenderness in the right upper quadrant with active bowel sounds. Murphy’s sign was negative with no significant guarding and rebound tenderness. The rest of the examination was unremarkable. The initial labs included white blood cell (WBC) count of 17.3 K/uL with 78% neutrophil, hemoglobin 11.8 g/dL, platelet 214 K/uL, hemoglobin A1c 6.5%, blood urea nitrogen (BUN)/creatinine 14/1.08 g/dL, alanine aminotransferase/aspartate aminotransferase (ALT/AST) 93/73 U/L, alkaline phosphatase (ALP)/total bilirubin (T Bili) 160/0.9 mg/dL, and C-reactive protein (CRP) 249 mg/L. Her urinalysis showed WBC >100/hpf, red blood cell 0-5, nitrite negative, leukocyte esterase large, hyaline cast 0-5, and rare bacteria. The coagulation profile was within normal limits. The hepatitis panel, gastrointestinal polymerase chain reaction panel, and stool for ova/parasite screen were non-significant. Urine and blood cultures were sent, and the patient was started empirically on ceftriaxone and metronidazole.

**Figure 1:**
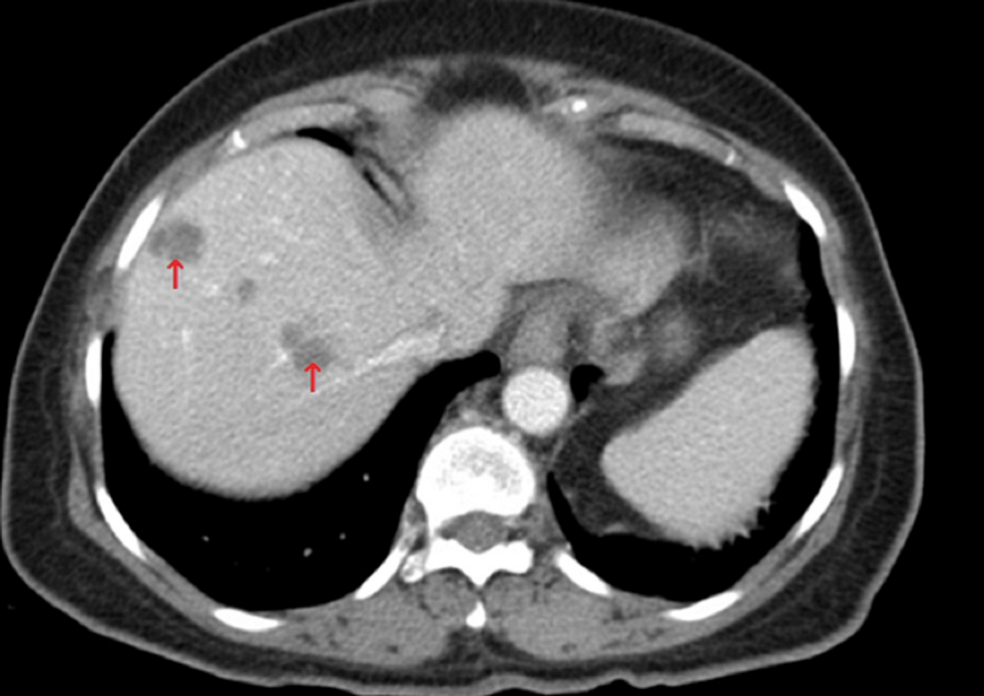
CT A/P axial view demonstrating multiple, hypoattenuating, ill-defined cystic lesions in the liver (red arrows) with peripheral enhancement and internal septation CT A/P: computed tomography of the abdomen and pelvis

Abdominal ultrasound was done, which suggested an echogenic lesion in the gallbladder wall measuring up to 0.3 cm, most likely a polyp. No liver masses were demonstrated. However, the CT scan of the abdomen/pelvis showed multiple hypo-attenuating, ill-defined lesions in the right hepatic lobes, with the largest lesion measuring up to 2.9 cm with a small amount of peripheral enhancement and internal septation (Figure 1). These findings were suggestive of early abscess formation in the liver versus metastasis.

**Figure 2:**
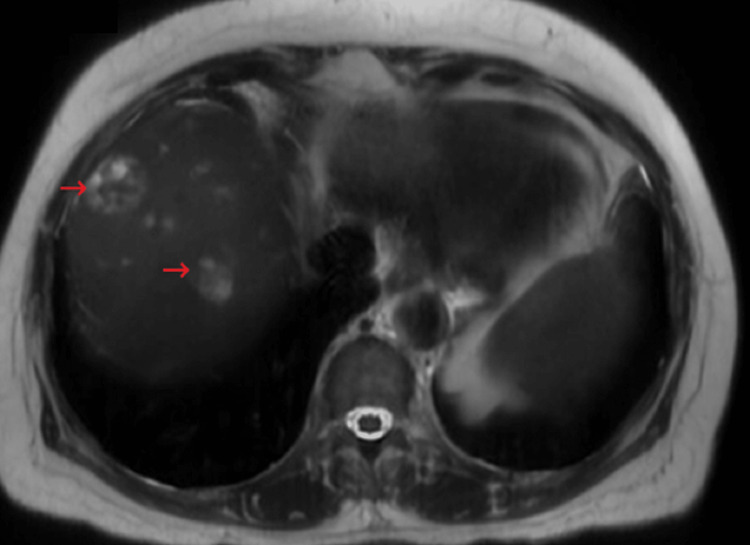
MRCP showing multiple hepatic abscesses (red arrows) with communicating intrahepatic biliary ductal dilatation, hyper-enhancement of the gallbladder wall, and a dampened signal filling the gallbladder/biliary tree MRCP: magnetic resonance cholangiopancreatography

The MRI of the liver demonstrated multiple hepatic abscesses with communicating intrahepatic biliary ductal dilatation, hyper-enhancing gallbladder wall, and relatively dampened signal filling the gallbladder/biliary tree. The MRCP confirmed these findings and was consistent with acute cholangitis and associated hepatic microabscesses (Figure 2).

A transthoracic echocardiogram revealed an ejection fraction of 55-60% and normal valve structures without any vegetation. Three days later, Streptococcus anginosus alpha hemolytic strep (sensitive to ceftriaxone, clindamycin, erythromycin, levofloxacin, penicillin, vancomycin) was isolated from the blood culture, while urine culture was negative. The repeat blood cultures obtained on the third and fourth day of admission were negative.

While hospitalized, ceftriaxone and metronidazole were continued. She continued to clinically improve and an improvement in lab parameters was notable during the course of her hospital stay. The patient was discharged on oral antibiotics (levofloxacin and metronidazole) after seven days of inpatient treatment with outpatient follow-up. She was asymptomatic with unremarkable laboratory values during her follow-up visit with the infectious disease specialist three weeks later (Table 1). She was then switched to oral clindamycin for six weeks duration.

**Table 1:** Laboratory testing revealed an initial leukocytosis, transaminitis, and elevated inflammatory markers with complete resolution after three weeks

Lab Test	Initial value	3-week value	Reference range
WBC	17.3 K/uL	5.7 K/uL	4.5-10.9 K/uL
ALT	93 U/L	14 U/L	0-31 U/L
AST	73 U/L	19 U/L	10-35 U/L
ALP	160 U/L	113 U/L	25-125 U/L
T Bili	0.9 mg/dL	0.3 mg/dL	0.0-1.2 mg/dL
BUN	14 mg/dL	11 mg/dL	8-23 mg/dL
Creatinine	1.08 gm/dL	0.9 mg/dL	0.5-0.9 mg/dL
CRP	249 mg/L	1.2 mg/L	1-4 mg/L

**Figure 3:**
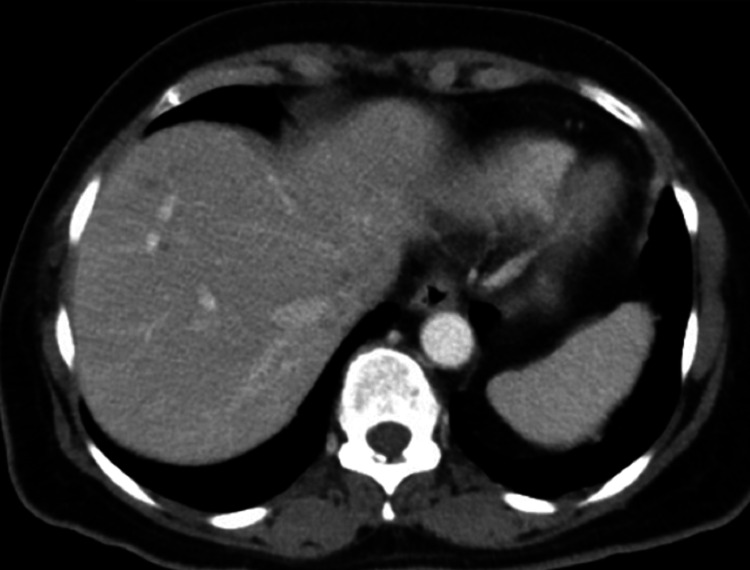
Repeat CT A/P showing near complete resolution of all hepatic lesions at approximately six weeks CT A/P: computed tomography of the abdomen and pelvis

In a six-week follow-up, the repeat CT scan showed complete resolution of all hepatic lesions, except for one small hypo-attenuating lesion (5.5 mm). This lesion also decreased in size compared to the previous finding of 2.9 cm (Figure 3). The patient was referred for a colonoscopy to rule out colon cancer.

## Discussion

Hepatic abscesses are rare disease entities, with an approximate annual incidence of 2.3 per 100,000 people [3], and the etiologic agent is usually polymicrobial [4]. Common agents include enteric gram-negative bacilli (Escherichia coli, Klebsiella pneumonia), Streptococcus milleri group (SMG), and anaerobes. Streptococcus species was identified as the culprit in 29.5% of pyogenic liver abscesses [5]. Streptococcus anginosus belongs to the “Streptococcus milleri group” of bacteria, which includes two other different species: Streptococcus intermedius and Streptococcus constellatus [6]. These bacteria reside as normal flora in the respiratory, gastrointestinal tract, and urogenital tract; and have the potential for abscess formation by virtue of their multiple virulence factors, including polysaccharide capsules, exotoxins, and hydrolytic enzymes like hyaluronidase and DNase [7].

Hepatic abscesses usually occur by one of the four routes: infection from the abdomen spreading through the portal vein, direct spread from a biliary source, hematogenous dissemination of any systemic infection, or penetrating wound [8]. Given the MRI finding of hepatic abscess communicating with intrahepatic biliary ductal dilation, the source was likely biliary in our patient. Although the MRI/MRCP was concerning for biliary cholangitis, she lacked clinical criteria for cholangitis (no fever and jaundice). Biliary tract disorders are most frequently associated with liver abscesses caused by SMG [9].

Our patient presentation was consistent with the common clinical features seen in a pyogenic liver abscess (fever/chills, nausea/vomiting, right upper quadrant pain, and elevated liver enzymes); however, these findings are not diagnostic of PLA [10]. Therefore, imaging modalities play a crucial role in establishing the diagnosis. Also, isolating organisms from either blood culture or abscesses is equally important in guiding the antibiotic regimen.

Streptococcus anginosus bacteremia would need further evaluation for deep-seated infection, including endocarditis and pyelonephritis [11]. The treatment usually involves two weeks of antibiotics if it is uncomplicated bacteremia. However, prolonged duration of therapy of about 6 weeks is needed in cases with abscesses as in our patient. Drainage, either percutaneous or surgical, is recommended in those with a large (>5 cm) hepatic abscess [12].

## Conclusions

In conclusion, a hepatic abscess is an uncommon condition that presents with common complaints like abdominal pain and fever. A high degree of suspicion with appropriate imaging can help with diagnosis. The ultrasound may not be sensitive enough to detect these lesions as seen in our patient. Further imaging with CT A/P and MRI/MRCP is needed for confirmation. As PLA is associated with significant mortality, timely management with appropriate antibiotics and/or aspiration/drainage is key to better outcomes.
